# Targeted Sequencing of the Mitochondrial Genome of Women at High Risk of Breast Cancer without Detectable Mutations in BRCA1/2

**DOI:** 10.1371/journal.pone.0136192

**Published:** 2015-09-25

**Authors:** Sophie Blein, Laure Barjhoux, GENESIS investigators, Francesca Damiola, Marie-Gabrielle Dondon, Séverine Eon-Marchais, Morgane Marcou, Olivier Caron, Alain Lortholary, Bruno Buecher, Philippe Vennin, Pascaline Berthet, Catherine Noguès, Christine Lasset, Marion Gauthier-Villars, Sylvie Mazoyer, Dominique Stoppa-Lyonnet, Nadine Andrieu, Gilles Thomas, Olga M. Sinilnikova, David G. Cox

**Affiliations:** 1 INSERM U1052, CNRS UMR5286, Université Lyon 1, Centre de Recherche en Cancérologie de Lyon, Lyon, France; 2 Inserm, U900, Paris, France; 3 Institut Curie, Paris, France; 4 Mines ParisTech, Fontainebleau, France; 5 Consultation de Génétique, Département de Médecine, Institut de Cancérologie Gustave Roussy, Villejuif, France; 6 Centre Catherine de Sienne, Nantes, France; 7 Institut Curie, Department of Tumour Biology, Paris, France; 8 Département de Cancérologie sénologique, CLCC Oscar Lambret, Lille, France; 9 Centre François Baclesse, Caen, France; 10 Oncogénétique Clinique, Hôpital René Huguenin/Institut Curie, Saint-Cloud, France; 11 Université Lyon 1, CNRS UMR5558, Lyon, France; 12 Unité de Prévention et d’Epidémiologie Génétique, Centre Léon Bérard, Lyon, France; 13 Institut Curie, INSERM U830, Paris, France; 14 Université Paris Descartes, Sorbonne Paris Cité, France; 15 Université Lyon 1, INCa-Synergie, Centre Léon Bérard, 28 rue Laennec, Lyon Cedex 08, France; 16 Unité Mixte de Génétique Constitutionnelle des Cancers Fréquents, Hospices Civils de Lyon - Centre Léon Bérard, Lyon, France; IFOM, Fondazione Istituto FIRC di Oncologia Molecolare, ITALY

## Abstract

Breast Cancer is a complex multifactorial disease for which high-penetrance mutations have been identified. Approaches used to date have identified genomic features explaining about 50% of breast cancer heritability. A number of low- to medium penetrance alleles (per-allele odds ratio < 1.5 and 4.0, respectively) have been identified, suggesting that the remaining heritability is likely to be explained by the cumulative effect of such alleles and/or by rare high-penetrance alleles. Relatively few studies have specifically explored the mitochondrial genome for variants potentially implicated in breast cancer risk. For these reasons, we propose an exploration of the variability of the mitochondrial genome in individuals diagnosed with breast cancer, having a positive breast cancer family history but testing negative for *BRCA1*/*2* pathogenic mutations. We sequenced the mitochondrial genome of 436 index breast cancer cases from the GENESIS study. As expected, no pathogenic genomic pattern common to the 436 women included in our study was observed. The mitochondrial genes *MT-ATP6* and *MT-CYB* were observed to carry the highest number of variants in the study. The proteins encoded by these genes are involved in the structure of the mitochondrial respiration chain, and variants in these genes may impact reactive oxygen species production contributing to carcinogenesis. More functional and epidemiological studies are needed to further investigate to what extent variants identified may influence familial breast cancer risk.

## Introduction

In 2008, 14 million breast cancers (BC) were diagnosed worldwide, representing more than 10% of all cancer diagnoses[1]. One woman out of nine will develop breast cancer in her lifetime in developed countries[1,2]. Breast cancer is a complex multifactorial disease, with genetic, life-style and environmental susceptibility factors. Today we estimate that the genetic component of breast cancer represents 5% to 10% of all breast cancers, with 4% to 5% due to high-penetrance mutations in susceptibility genes[3–5] Mutations in two high-penetrance susceptibility genes have been identified: *BRCA1* and *BRCA2*. Pathogenic mutations in *BRCA1* confer a lifetime risk of BC of 60% to 85%[6,7], whereas such mutations in *BRCA2* confer a lifetime risk of approximately 40% to 85%[6,7]. Other genomic variations (for example in genes interacting with *BRCA1* and *BRCA2*) have been identified as modifiers of BC cancer risk and alter the risk initially conferred by *BRCA1* or *BRCA2* mutation[8].

Early methods such as linkage or candidate gene studies, and more recent approaches like genome-wide association studies (GWAS) have been able to identify genomic features explaining about 50% of breast cancer heritability[9]. A number of low- to moderate-effect Single Nucleotide Polymorphisms (SNPs) have been identified as associated with BC risk. However, today the large amount of genome-wide studies performed that have failed to identify other highly penetrant breast cancer susceptibility genes suggests that the remaining heritability is likely to be explained by the cumulative effect of low-penetrant and low- to moderate-effect genomic variations, by moderately penetrant but rare alleles, and by the synergistic effect of environmental exposure combined to specific genomic variations.

Oxidative stress has been shown to play a role in BC development[10,11]. Mitochondria are intimately linked to oxidative stress, as they are a main source of Reactive Oxygen Species (ROS) in the cell. ROS are cytotoxic and mutagenic components that can cause damage to DNA, in particular double strand breaks. When not correctly handled, this damage leads to genomic instability, and therefore may contribute to tumorigenesis. Germline and somatic variations in the mitochondrial genome have been linked to cancer. Mitochondrial genome mutations are found in various cancers and may alter mitochondrial metabolism, enhance tumorigenesis and permit cancer cell adaptation to changing environments[12,13].

Whereas the mitochondrial genome is haploid, classical linkage studies are dedicated to the analysis of diploid genomes. Furthermore, commercially available GWAS arrays are not suited to capture variation in specific regions of the genome, in particular the mitochondrial genome. Therefore methods specifically targeting the mitochondrial genome are needed to further explore variability in the mitochondrial genome of breast cancer patients. For these reasons, we have sequenced the mitochondrial genome of 436 women diagnosed with breast cancer, having a positive familial breast cancer history, but testing negative for *BRCA1/2* pathogenic mutations.

## Materials and Methods

### GENESIS study

GENESIS (GENE SISters) is a French national study designed to identify new breast cancer susceptibility genes[14]. GENESIS includes pairs of sisters diagnosed with breast cancer, but testing negative for *BRCA1/BRCA2* mutation. Testing was performed in the context of an oncogenetic consultation following French national guidelines. GENESIS also includes matched non-related controls of the same age group and sharing the same living environment, i.e. close friends or colleagues of index cases.

### Ethics Statement

The GENESIS study has been reviewed and approved by the appropriate ethics committee (Comité de Protection des Personnes Ile de France III, 3 october 2006, agreement n°2373). Informed consent has been obtained for each woman included in the GENESIS Study.

### Sample Selection

We selected unrelated women among index breast cancer cases of the study. Index cases were ranked by breast cancer phenotypic aggressiveness: *i*.*e*. women diagnosed at an early age, and presenting bilateral cancer (see Table 1). Table 1 also presents the number of cases selected from different inclusion categories. Blood samples are centralized in the Breast Cancer Genetics laboratory of the Cancer Research Center of Lyon, where DNA is extracted using automated protocols. As our hypothesis was that rare, potentially deleterious alleles would be discovered, we chose to include as many index cases as possible, and no control individuals. Statistical power to carry out classical association testing in this context would be insufficient, and formal association testing was not the aim of this study.

**Table 1:** Inclusion categories of selected index cases.

Inclusion category	Counts	
Index Case’s BC bilateral, diagnosed before 50 Sister’s BC bilateral, diagnosed before 50	8	
Index Case’s BC bilateral, diagnosed before 60 Sister’s BC bilateral	8	
Index Case’s BC diagnosed before 60 Sister’s BC bilateral	36	
Index Case’s BC bilateral, diagnosed before 50 Sister’s BC unilateral, diagnosed before 50	23	
Index Case’s BC unilateral, diagnosed before 50 Sister’s BC bilateral, diagnosed before 50	23	
Index Case’s BC unilateral, diagnosed before 50 Sister’s BC unilateral, diagnosed before 50	164	
Index Case’s BC bilateral, diagnosed before 60	174	
**Total**	**436**	
**Age at 1st BC for index cases** (in years, mean ± SD)	46.1 ± 7.9	
**Age at 1st BC for sister** (in years, mean ± SD)	45.7 ± 6.7	
**Bilateral cancer for index cases**	Yes: 51.2%	No: 48.8%

### Mitochondrial Genome Sequencing

Primers corresponding to eleven amplicons covering the whole mitochondrial genome were designed and tested for specificity to the mitochondrial genome (Table A in S1 File). Sequencing libraries were prepared with NEBNext Fast DNA Fragmentation & Library Prep Set for Ion Torrent. The mean target fragment size for fragmentation was 175 bp. Samples were multiplexed by 48. Libraries were loaded on Ion 316 chips for sequencing (Single end sequencing) using the Personalized Genome Machine^TM^ system (PGM, Life Technologies, Carlsbad, CA, USA). A total of 10 sequencing runs were performed.

### Bioinformatics Analyses

Reads were aligned to the Mitochondrial Genome Reference sequence rCRS deposed in Genbank under the accession NC_012920.1 with BWA[15] software (version 0.7.5a). Read quality was assessed using the FastQC (v.0.10.1)[16] Toolkit. Samtools (v.0.1.19)[17], Picard tools (v.1.96)[18], and GATK (v.2.5–2)[19–21] were used to process aligned bam files. Indels were realigned after primary alignment with the GATK walker IndelRealigner. Base quality scores were recalibrated with the GATK walker BaseRecalibrator. Variants were called using the GATK UnifiedGenotyper algorithm. Visual examination of read alignment in regions surrounding detected variants was performed with Integrative Genomics Viewer[22,23].

### Annotation and filtering

Variants called were annotated with the Ensembl tool Variant Effect Predictor (VEP)[24], and with locally developed Python scripts. Given the haploid status of mitochondria, variants called as heterozygous were considered unreliable and filtered as false-positives. Variants for which the alternative allele was not balanced on both strands, *i*.*e*. variants having less than 10% of reads supporting alternative allele on one strand are considered as unreliable and filtered. Remaining reliable variants were annotated according to MITOMAP[25], a catalog of mitochondrial variants including mini insertions and deletions (accessed September 2013). We also annotated positions that are conserved among vertebrates, i.e. positions strictly conserved between reference genomes of nine superior eukaryotic species (data not shown). If mutated, variants at these positions are potentially more likely to have a functional impact. Finally, VEP provided gene annotation, substitution effect in transcribed region, codon and amino acid change and functional effect prediction with PolyPhen[26] and SIFT[27] for non-synonymous substitutions.

To estimate mitochondrial variability at the gene level, 2 statistics were computed. The global variant enrichment rate estimates the number of distinct variants observed per gene and per Mb. For a given gene *x* of length *l*_*x*_, if *N*_*x*_ distinct variants were observed in our study, then the global variant enrichment rate *r*_*g*_ for this gene is:

rg = Nxlx ×  1000 variants/Mb

This statistic does not take into account the frequency of each variant observed in our study. Therefore, the weighted variant enrichment rate *r*_w_, taking into account both gene variability and variants frequency was also computed. With *i* from 1 to *N*_*x*_ representing the *i* ^th^ variant observed on gene *x*, and *n*_*i*_ the count of individual carrying variant *i*, then:

$$r_{w}\;=\;\frac{\Sigma_{i=1}^{N_{x}}n_{i}}{l_{x}}\;\;\times \;\;1000\;variants.person/Mb$$

The statistics were computed for all mitochondrial genes, except for those encoding for mitochondrial transfer RNAs, as their very small length would have biased the results.

## Results

### Sequencing characteristics, coverage assessment, and variant description

More than 20 million reads were obtained, with a mean read count per sample higher than 45,000 (Table B in S1 File). On average, more than 27,000 of the 45,000 sequenced reads were successfully aligned against the reference genome, which gives an alignment rate of 57.4%. (Table C in S1 File)

In our experiment, approximately 90% of the mitochondrial genome is covered with a depth of coverage of 50X. The global mean coverage is 198X. More details, including coverage profile along the mitochondrial genome, are presented in Figure A in S1 File.

1157 distinct variants successfully passed our quality filters; and are detailed in S2 File. More than 99% of variants are substitutions. About 80% of variants are located in the coding sequence of mitochondrial genes. This is not surprising given the probable bacterial origin of mitochondria, and the low proportion of intergenic sequences in its genome. There are no missense substitutions or indels detected in genes coding for mitochondrial transfer RNA (Mt-tRNA). Comparing concordance between analyses with SIFT and Polyphen shows that 24 variants are predicted as *deleterious* by SIFT and as *probably damaging* by Polyphen, and these variants are detailed in Table 2.

**Table 2:** Description of variants predicted as deleterious by SIFT and as probably damaging by Polyphen.

Position	Ref.	Alt.	rsID	MITOMAP	Counts	Conserved	Gene	CodonChange	a.a Change
3388	C	A	.	Known	1	No	MT-ND1	Cta/Ata	L/M
6237	C	A	.	Known	2	No	MT-CO1	Ctg/Atg	L/M
6489	C	A	rs28461189	Known	3	Yes	MT-CO1	Ctc/Atc	L/I
7941	A	G	.	Known	1	Yes	MT-CO2	aAc/aGc	N/S
7964	T	C	.	Known	1	No	MT-CO2	Ttc/Ctc	F/L
7976	G	A	.	Known	1	Yes	MT-CO2	Ggc/Agc	G/S
8563	A	G	.	Known	1	No	MT-ATP6	Aca/Gca	T/A
8839	G	A	.	Known	2	Yes	MT-ATP6	Gcc/Acc	A/T
8920	G	A	.	Known	1	No	MT-ATP6	Ggc/Agc	G/S
9010	G	A	.	Known	1	Yes	MT-ATP6	Gct/Act	A/T
9448	A	G	.	Known	1	Yes	MT-CO3	tAc/tGc	Y/C
9500	C	A	.	Unknown	1	No	MT-CO3	ttC/ttA	F/L
9577	T	C	.	Unknown	1	Yes	MT-CO3	cTa/cCa	L/P
9903	T	C	rs199999390	Known	1	Yes	MT-CO3	Ttt/Ctt	F/L
11087	T	C	.	Known	1	Yes	MT-ND4	Ttc/Ctc	F/L
12634	A	G	.	Known	3	Yes	MT-ND5	Atc/Gtc	I/V
12923	G	T	.	Known	1	No	MT-ND5	tGa/tTa	W/L
13129	C	T	.	Known	1	Yes	MT-ND5	Ccc/Tcc	P/S
13973	A	T	.	Known	1	No	MT-ND5	cAa/cTa	Q/L
14180	T	C	.	Known	2	No	MT-ND6	tAt/tGt	Y/C
14484	T	C	.	Known	2	No	MT-ND6	Atg/Gtg	M/V
14769	A	G	rs28357679	Known	2	Yes	MT-CYB	aAc/aGc	N/S
15218	A	G	rs2853506	Known	10	No	MT-CYB	Aca/Gca	T/A
15773	G	A	.	Known	1	Yes	MT-CYB	Gta/Ata	V/M

Ref.: Reference allele

Alt.: Alternative allele

Freq.: Minor allele frequency observed in our data

a. a. change: amino acid change

Table 3 contains a description of observed variants unknown in MITOMAP, which represent 3% of all detected variants. None of these variants is detected at high frequency in our dataset (all were observed once, with the exception of the A-T variant at bp3385 which was observed twice), and most do not affect conserved positions. These observations are consistent with unknown rare polymorphisms, and these variants are also absent from dbSNP.

**Table 3:** Description of variants observed but unknown in MITOMAP.

Pos.	Ref.	Alt.	Conserved	Gene	Effect	Sift	Polyphen
393	T	A	No	-	-	-	-
1713	A	G	No	MT-RNR2	Non coding exon	-	-
1807	T	C	No	MT-RNR2	Non coding exon	-	-
2150	T	TA	Yes	MT-RNR2	Non coding exon	-	-
2156	A	AT	No	MT-RNR2	Non coding exon	-	-
3385	A	T	No	MT-ND1	missense	**deleterious**	**possibly damaging**
4875	C	T	No	MT-ND2	synonymous	-	-
5573	A	G	No	MT-TW	Non coding exon	-	-
5746	GA	G	No	-	-	-	-
6113	A	T	No	MT-CO1	synonymous	-	-
6200	C	T	No	MT-CO1	synonymous	-	-
6569	C	T	No	MT-CO1	synonymous	-	-
6608	C	T	No	MT-CO1	synonymous	-	-
6812	A	G	No	MT-CO1	synonymous	-	-
7004	A	G	No	MT-CO1	synonymous	-	-
7366	C	T	Yes	MT-CO1	missense	tolerated	benign
8263	C	T	No	MT-CO2	synonymous	-	-
8465	C	T	No	MT-ATP8	missense	tolerated	**probably damaging**
8673	A	G	No	MT-ATP6	synonymous	-	-
9138	C	T	No	MT-ATP6	synonymous	-	-
9370	A	T	No	MT-CO3	missense	tolerated	benign
9500	C	A	No	MT-CO3	missense	**deleterious**	**probably damaging**
9577	T	C	Yes	MT-CO3	missense	**deleterious**	**probably damaging**
9873	C	A	No	MT-CO3	missense	tolerated	**probably damaging**
9890	A	G	No	MT-CO3	synonymous	-	-
10030	C	T	No	MT-TG	Non coding exon	-	-
10094	C	A	No	MT-ND3	synonymous	-	-
12098	C	T	No	MT-ND4	synonymous	-	-
12266	A	G	No	MT-TL2	Non coding exon	-	-
13380	C	T	No	MT-ND5	synonymous	-	-
13792	C	T	No	MT-ND5	synonymous	-	-
13806	C	T	No	MT-ND5	synonymous	-	-
15620	C	T	Yes	MT-CYB	missense	**deleterious**	**possibly damaging**
16229	T	A	No	-	-	-	-
16454	C	T	No	-	-	-	-

Pos.: position on mitochondrial genome

Ref.: Reference allele

Alt.: Alternative allele

Table 4 summarizes the distribution of variants by mitochondrial gene, and displays the global variant enrichment rate *r*_*g*_ and the weighted enrichment rate *r*_*w*_ for each gene. The mean global variant enrichment per gene (Mt-tRNA genes excluded) is 64.2 variants per 1Mb. The mean weighted variant enrichment rate is 0.44 variants.person/Mb. Genes with the highest enrichment are *MT-ATP6* with 86.5 variants/Mb, and *MT-CYB* (cytochrome B) with 84.1 variants/Mb. A Shapiro test was used to verify that *r*_*g*_ follows a normal distribution (p-value = 0.34). *r*_*g*_ values of *MT-ATP6* and *MT-CYB* are positioned at the 93^th^ and at the 90^th^ percentile of the distribution. They are also among genes having the highest weighted variant enrichment rate (0.92 and 0.91 respectively).

**Table 4:** Distribution of variants and description by mitochondrial gene. Variant Enrichment is performed for non Mt-tRNA genes (Variants per 1Mb).

Gene Symbol	Type	Length	Variant count	Enrichment *r*_*g*_	Enrichment *r*_*w*_
MT-RNR1	Mt_rRNA	955	34	35.6	1.07
MT-RNR2	Mt_rRNA	1561	57	37.2	0.64
MT-ND1	protein_coding	957	58	60.6	0.27
MT-ND2	protein_coding	1043	64	61.4	0.62
MT-CO1	protein_coding	1543	98	63.5	0.29
MT-CO2	protein_coding	685	44	64.2	0.11
MT-ATP8	protein_coding	208	15	72.1	0.12
MT-ATP6	protein_coding	682	59	86.5	0.92
MT-CO3	protein_coding	785	60	76.4	0.23
MT-ND3	protein_coding	347	24	69.2	0.30
MT-ND4L	protein_coding	298	20	67.1	0.23
MT-ND4	protein_coding	1379	85	61.6	0.46
MT-ND5	protein_coding	1813	134	73.9	0.27
MT-ND6	protein_coding	526	26	49.4	0.24
MT-CYB	protein_coding	1142	96	84.1	0.91

*r*_*g*_: global variants enrichment rate, in variants/Mb.

*r*_*w*_ weighted variants enrichment rate, in variants.person/Mb

Two genes, MT-RNR1 and MT-RNR2, show low global enrichment rates (35.6 and 37.2 variants/Mb, respectively). These two genes code for the mitochondrial 12S and 16S ribosomal RNAs, respectively structural components of the small and large ribosomal subunits of mitoribosomes. Given their essential structural function, it is not surprising to see that these genes are more conserved than other mitochondrial genes.

**Fig 1:**
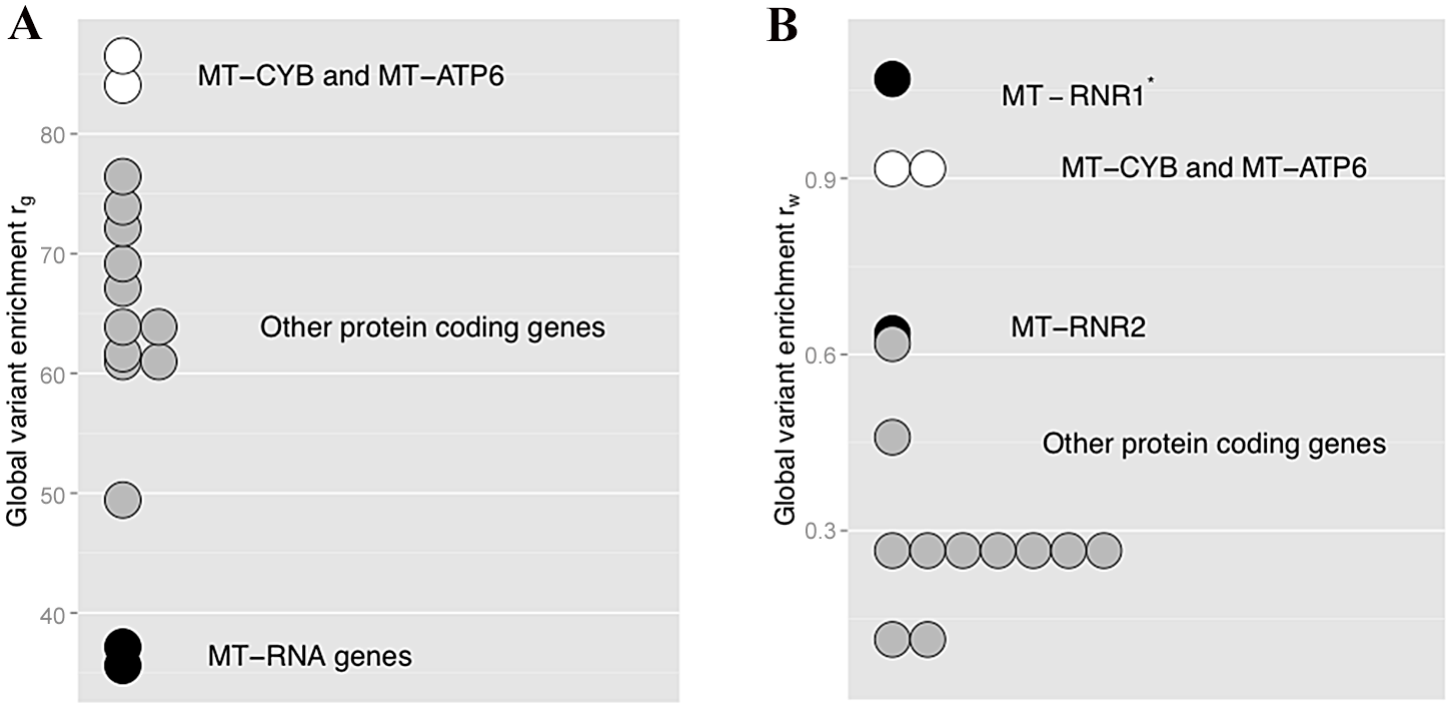
Distribution of *r*_*g*_ and *r*_*w*_ for all non-MT-tRNAs genes. A. Distribution of ***r***_***g***_ by bins of 1. B. Distribution of ***r***_***w***_ by bins of 1. White dots represents *MT-CYB* and *MT-ATP6*. Black dots represents *MT-RNR1* and *MT-RNR2*. Grey dots represent other protein coding genes. * ***r***_***w***_ value for *MT-RNR1* gene is artificially increased because of 2 rare variants carried in the reference sequence in this gene.

As represented in Fig 1, we can observe 3 groups of genes. *MT-RNR1* and *MT-RNR2* represent the group of MT-RNAs genes. They are characterized by a low number of distinct variants, some having a high frequency in our population. On the other hand, *MT-CYB* and *MT-ATP6* are both characterized by a high number of distinct variants (high *r*_*g*_ value). They also are the most frequently altered genes in our population (high *r*_*w*_ value). The third group is composed of the remaining genes.

## Discussion

In this study we have performed a deep characterization of mitochondrial genome variability among 436 women diagnosed with breast cancer, having a positive familial breast cancer history, and negative for *BRCA1/2* mutation screening. Women were chosen based on the age at diagnosis and on the laterality of their cancer.

We identified 1157 distinct variants compared to the reference sequence rCRS. All the frequent variants observed in this study (*i*.*e*. with a relative frequency > 5%) are known in the MITOMAP database. The majority of these variants are common mitochondrial SNPs, some of which have been associated with an increased risk of breast cancer[28, 29]. Of the 35 variants (including 3 indels) that are unknown in MITOMAP database, (also unknown in dbSNP), none was observed in more than 3 individuals among the 436 samples that were sequenced. There is no reason to reject these variants after visual examination of alignments with IGV. They could be rare variants or private mutations, *i*.*e*. rare mutations restricted to a few families, appeared recently in mitochondrial genome evolution. 4 variants are predicted as *deleterious* by SIFT, 2 are predicted *possibly damaging*, and 4 *probably damaging* by PolyPhen. None of these 6 loci have been described in the literature.

No variant predicted as *deleterious* by SIFT and *probably damaging* by PolyPhen was observed more than 3 times in the 436 samples sequenced, except the polymorphism rs2853506 (A15218G), which was observed in 10 samples (2%). This polymorphism has been found associated with epileptogenesis[30]. This SNP is located in the gene coding for cytochrome b, *MT-CYB*. Along with cytochrome c1 and Rieske protein, cytochrome b is one of the three respiratory subunits of complex III, the third complex involved in Electron Transport Chain. Alterations in this subunit have been shown to modify catalytic capacities of complex III[31].

Although the coverage profile is not homogeneous or constant along the mitochondrial genome, this profile is robust through the 10 independent sequencing runs that were performed in this study. Furthermore, despite the relatively low alignment rate, we have a mean depth > 50X for approximately 90% of mitochondrial genome. Similar coverage profiles were obtained in other studies on Ion Torrent sequencing platforms and with various aligners[32,33].

Whereas the Ion Torrent sequencing technology has been reported to have very high confidence rates regarding point mutation sequencing, the reliability of detecting insertions and deletions (indels) has been a subject of intense debate and a significant proportion of indels called with this technology is likely to represent false positives [33–35]. In our study, only 10 out of 111 indels initially called passed filters, and 93% of these were discarded in at least one sample due to an unbalanced repartition of reads supporting alternative alleles on the two DNA strands, with less than 10% of reads on one strand. This strand specific deletion bias has already been observed in Ion Torrent [36]. Furthermore, in our results, 8 out of the 10 deletions that have passed filters are located in homopolymer tracts. The only 2 remaining indels seem highly reliable when visualized with a viewer like IGV[22,23]. Ion Torrent sequencing is known to be prone to errors for homopolymeric tracts of length more than 3, the uncertainty of pH measure often leading to a misestimation of homopolymer length. Loman *et al*. have estimated the Ion Torrent PGM homopolymer-associated indel errors at 1.5 per 100 bases[37]. Better results are usually obtained on Ion Torrent PGM data by using an aligner based on Burrows-Wheeler Transform (BWT) algorithm[38], an alignment algorithm used by BWA (the aligner we used) or the mapper included in Nextgene sequencing analysis software (SoftGenetics, State College, PA, USA).

Filtering non-homozygous variant calls enables us to obtain a restricted list of variants after having eliminated the high level of noise generated by Ion torrent sequencing. However, by doing this we choose to not analyze the potential heteroplasmy, or potential somatic changes in the mitochondrial genome in the blood sample sequenced, and to focus on inherited variants. We did so because heteroplasmy in blood may not be of interest for studying breast cancer etiology, as compared to heteroplasmy observed in breast tissue.

Our aim is to characterize in detail mitochondrial genome variability of women with a strong familial history of breast cancer, but without *BRCA1/2* pathogenic mutations. Given the diminished capability of familial linkage and GWAS analyses to detect mitochondrial genome features, a targeted sequencing approach was needed. Several recent studies[38] underline the possibility that the remaining unexplained heritability of breast cancer could be explained by rare variants specific of only one or a few families. Population based methods like case-control studies are therefore not able to detect any associations with these variants. For this reason we have not included any control subjects in our study, and have included as many cases as feasible.

We identified 1157 variants; some of which were previously associated with breast cancer risk. However, as expected, we did not identify a common potentially pathogenic genomic pattern among the 436 women included in our study. It is therefore unlikely that mutations in the mitochondrial genome have the potential to explain a high proportion of the excess of breast cancer risk of women with a familial history but without *BRCA1/2* mutations. New variants, unreferenced in MITOMAP and not described in the pubic literature were characterized, and some of them are predicted to potentially alter the function of the protein encoded by their genes. *MT-ATP6* and *MT-CYB*, two genes coding for important structural subunits of the mitochondrial respiration chain, show the highest variability with the highest frequency in our data. These results are interesting in this context, and echo recent results underlying the potential role of mitochondrial variants as susceptibility factors for familial breast cancer risks[39]. Based on these elements, further studies are needed to examine if these variants influence breast cancer risk.

Alternative approaches are now required to try to understand a larger part of breast cancer heritability. Powerful technologies such as next generation sequencing can help to achieve this goal, and has already been applied in the context of breast cancer[40,41]. New consortiums are emerging, such as COMPLEXO which aims at deciphering breast cancer missing heritability by looking further into the human exome by using massive parallel sequencing technologies[42]. Our results provide evidence that the mitochondrial genome should be considered when designing studies in order assess the role of mitochondrial genome variability in breast cancer risk and etiology.

## Supporting Information

S1 File Technical details of sequencing experiments. Details of PCR primers used to isolate the mitochondrial genome including primer sequence, melting temperature, and amplicon size (**Table A**). Post-sequencing read characteristics (**Table B**). Post-sequencing mitochondrial genome coverage (**Table C**). Coverage distribution along the mitochondrial genome with standard deviation for all 436 samples. Coverage in overlapping regions is divided by two to take into account the overlap (**Figure A**).
(DOCX)

S2 File Full list of variants identified. Details of all variants identified by sequencing 436 mitochondrial genomes (**Table A**).
(XLSX)
