# Supplementary material for: Targeted Sequencing of the Mitochondrial Genome of Women at High Risk of Breast Cancer without Detectable Mutations in BRCA1/2
Source: PLoS One. 2015 Sep 25;10(9):e0136192. doi: 10.1371/journal.pone.0136192 (PMC4583250; doi:10.1371/journal.pone.0136192)

**Supplementary Materials**

**Table A: PCR details**

| **Primer Sequence** | **Tm** | **Name** | **Length** | **Amplicon Length (bp)** |
| --- | --- | --- | --- | --- |
| 5'- TCA CGG GAG CTC TCC ATG CAT -3' | 60.9 | Mi1F | 21 | 1651 |
| 5'- TAG GTT TAG CTC AGA GCG GTC AAG -3' | 58.3 | Mi1R | 24 |  |
| 5'- CTG GAA AGT GCA CTT GGA CGA A -3' | 57.7 | Mi2F | 22 | 1751 |
| 5'- AGT AGG AGG TTG GCC ATG GGT ATG -3' | 60.4 | Mi2R | 24 |  |
| 5'- GAG CCC GGT AAT CGC ATA AAA CTT -3' | 57,6 | Mi3F | 24 | 1690 |
| 5'- AGA AGG CTT ACG TTT AGT GAG GGA G -3' | 58 | Mi3R | 25 |  |
| 5'- TCT GAC ATC CGG CCT GCT TCT -3' | 60.8 | Mi4F | 21 | 1640 |
| 5'- CTG TGA TTA GGA CGG ATC AGA CGA -3' | 57.8 | Mi4R | 24 |  |
| 5'- GTG TCT CCT CTA TCT TAG GGG CCA TC -3' | 59.7 | Mi5F | 26 | 1733 |
| 5'- TAA GCC TAA TGT GGG GAC AGC TCA T -3' | 59.8 | Mi5R | 25 |  |
| 5'- ACG TTG ACA ATC GAG TAG TAC TCC CGA -3' | 60.5 | Mi6F | 27 | 1783 |
| 5'- TCG GAA ATG GTG AAG GGA GAC T -3' | 57.9 | Mi6R | 22 |  |
| 5'- TCA GGA GTA TCA ATC ACC TGA GCT CA -3' | 58.9 | Mi7F | 26 | 1707 |
| 5'- CAG GAG TTT GAT AGT TCT TGG GCA GTG -3' | 59.3 | Mi7R | 27 |  |
| 5'- CTC CCT TCC CCT ACT CAT CGC A -3' | 60.4 | Mi8F | 22 | 1738 |
| 5'- GCT TGG ATT AGC GTT TAG AAG GGC T -3' | 59.2 | Mi8R | 25 |  |
| 5'- CTA TAC AAC CGT ATC GGC GAT ATC GGT -3' | 59.4 | Mi9F | 27 | 1725 |
| 5'- ATT GTT AGC GGT GTG GTC GGG T -3' | 61.2 | Mi9R | 22 |  |
| 5'- AGC CAT CGC TGT AGT ATA TCC AAA G -3' | 56.4 | Mi10F | 25 | 1685 |
| 5'- TGG TAC CGT ACA ATA TTC ATG GTG GCT G -3' | 60.2 | Mi10R | 28 |  |
| 5'- GAA GCA GAT TTG GGT ACC ACC CAA G -3' | 59.8 | Mi11F | 25 | 679 |
| 5'- CAG ATA CTG CGA CAT AGG GTG CT -3' | 58.5 | Mi11R | 23 |  |

**Table B: Post-sequencing read characteristics**

| Total read count | 20,514,117 |
| --- | --- |
| Raw read count per sample *(mean ± sd )* | 47,050.73 ± 16,656.96 |
| Mapped read count per sample *(mean ± sd )* | 27,452.83 ± 10,703.89 |
| Raw Read length *(bp, mean ± sd )* | 136.8934 ± 55.49467 |

**Table C: Post-sequencing mitochondrial genome coverage**

| **Coverage threshold *T*** | | **Proportion of mtGenome covered less than *T* *(%, mean ± sd )*** |
| --- | --- | --- |
| 5 X | 0.3 ± 1.9 |  |
| 10 X | 0.8 ± 3.7 |  |
| 20 X | 2.4 ± 7.0 |  |
| 50 X | 10.7 ± 13.3 |  |

Figure A: Coverage Distribution along mitochondrial Genome.

Mean +/- Standard deviation over the 436 samples

Coverage in overlapping amplicon regions is divided by a factor 2


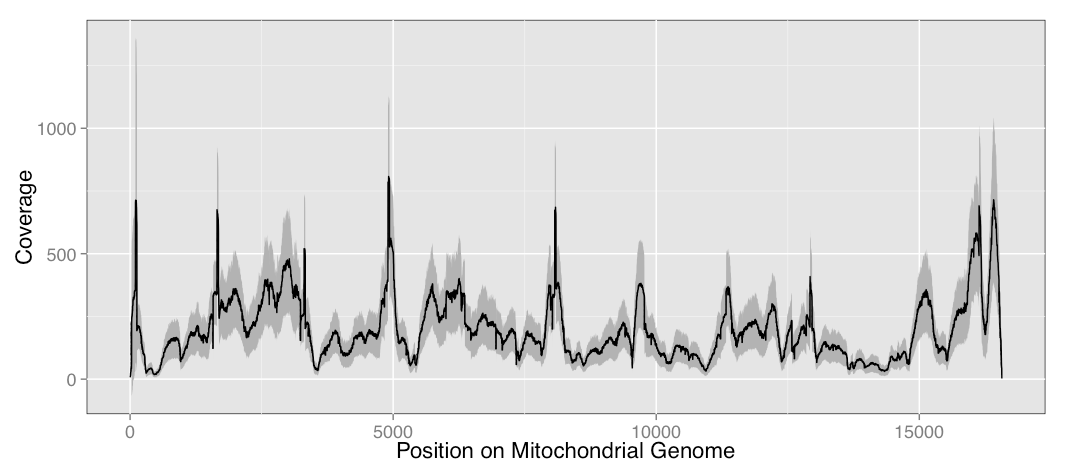

Supplement: S1 File — Details of PCR primers used to isolate the mitochondrial genome including primer sequence, melting temperature, and amplicon size (Table A). Post-sequencing read characteristics (Table B). Post-sequencing mitochondrial genome coverage (Table C). Coverage distribution along the mitochondrial genome with standard deviation for all 436 samples. Coverage in overlapping regions is divided by two to take into account the overlap (Figure A). (DOCX) [file pone.0136192.s001.docx]
